# Does Rearing Laying Hens in Aviaries Adversely Affect Long-Term Welfare following Transfer to Furnished Cages?

**DOI:** 10.1371/journal.pone.0107357

**Published:** 2014-09-17

**Authors:** Fernanda M. Tahamtani, Tone Beate Hansen, Rachel Orritt, Christine Nicol, Randi O. Moe, Andrew M. Janczak

**Affiliations:** 1 Animal Welfare Research Group, Department of Production Animal Clinical Science, NMBU, Oslo, Norway; 2 Animalia, Norwegian Meat and Poultry Research Centre, Oslo, Norway; 3 School of Psychology, University of Lincoln, Lincoln, United Kingdom; 4 Division of Animal Health and Husbandry, University of Bristol, Bristol, United Kingdom; Institut Pluridisciplinaire Hubert Curien, France

## Abstract

This study tests the hypothesis that hens that are reared in aviaries but produce in furnished cages experience poorer welfare in production than hens reared in caged systems. This hypothesis is based on the suggestion that the spatial restriction associated with the transfer from aviaries to cages results in frustration or stress for the aviary reared birds. To assess the difference in welfare between aviary and cage reared hens in production, non-beak trimmed white leghorn birds from both rearing backgrounds were filmed at a commercial farm that used furnished cage housing. The videos were taken at 19 and 21 weeks of age, following the birds' transition to the production environment at 16 weeks. Videos were analysed in terms of the performance of aversion-related behaviour in undisturbed birds, comfort behaviour in undisturbed birds, and alert behaviour directed to a novel object in the home cage. A decrease in the performance of the former behaviour and increase in the performance of the latter two behaviours indicates improved welfare. The results showed that aviary reared birds performed more alert behaviour near to the object than did cage reared birds at 19 but not at 21 weeks of age (*P* = 0.03). Blood glucose concentrations did not differ between the treatments (*P*>0.10). There was a significant difference in mortality between treatments (*P* = 0.000), with more death in aviary reared birds (5.52%) compared to cage birds (2.48%). The higher mortality of aviary-reared birds indicates a negative effect of aviary rearing on bird welfare, whereas the higher duration of alert behavior suggests a positive effect of aviary rearing.

## Introduction

Following the EU Council Directive ban on conventional ‘battery’ cages (99/74/EC) which came into full effect in January 2012 [1], concerns for the welfare implications of certain rearing and production system combinations have arisen. Previous studies indicate that rearing conditions affect the welfare of birds in the producing stage. Nicol et al. [2] found that previous exposure to wood shavings facilitated dust bathing behaviour in later life. However, it was also noted that current substrate provision (i.e. the substrate to which the birds had access at the time of observation) was more important than previous exposure with regards to effects on behaviour performed in the adult stage. Similarly, Wichman and Keeling [3] found that dust bathing behaviour was affected to a lesser degree by early rearing environment than current substrate access. Both studies suggest that adult birds are capable of adapting to their current environment, but that the speed at which this is achieved could be influenced by rearing conditions.

Rearing experience has also been found to effect production. Feather pecking, the non-aggressive pulling of feathers of other individuals, is influenced by early rearing [4], [5] and, if developed, increases mortality and the feed conversion ratio [6]. Studies report that hens reared in cages produced heavier eggs compared to aviary hens [7] and floor rearing yields dirty and cracked eggs more frequently than cage rearing [8]. However, hens reared in enriched environments had better performance against *Eimeria* and infectious Bronchitis [9]. Whatever type of laying accommodation is used, the ability of the birds to adapt to it will depend, to some extent, on their previous rearing experience.

Norwegian regulations specify that all birds must be reared in systems that provide dust bathing substrate and perching opportunities, including resources similar to those that the birds will have access to when they are housed in furnished cages during the production period. Due to the lack of rearing cages on the market that satisfy these requirements, this legislation implies that all chickens should be reared in aviaries irrespective of whether they will later produce in aviaries or furnished cages. Producers using furnished cage systems are concerned that adaptation to the more spatially restrictive environment of the furnished cage after rearing in aviaries may cause welfare problems for the birds. Reduced welfare in this context may result from frustration, or stress caused by exposure to environmental change. These mechanisms are not mutually exclusive, and are likely to exacerbate stress caused by transport from the rearing to the production farm and other physiological changes associated with the start of lay at approximately 18 weeks of age. In this context, frustration is related to the omission of an expected reward [10] in the form of a rich foraging substrate and limited restriction of movement. The concept of frustration is based upon the assumption that birds can form expectations. Frustration in laying hens has previously been measured by increases in the gakel-call frequency [11] and studied in relation to feather pecking behaviour [10]. Our previous studies firmly establish that chickens form expectations and that these can be quantified via changes in behaviour [12]–[15]. These studies underpin the consensus that frustration is probably experienced by laying hens.

Several aspects of transfer from one environment to another, in this case from rearing farm to producing farm, are known to cause stress. These factors include handling and increased human contact, changes in social structure, transportation, food and water deprivation, changes in climatic condition, physical injury and exposure to novel environments [16], [17]. These sources of stress have effects not only on animal welfare but also on animal product quality [16], [17]. One of the results of stress and associated activation of the hypothalamic-pituitary-adrenocortical (HPA) axis is secretion of catecholamines and glucocorticoids, which induce an increase in blood glucose concentrations to sustain fight or flight responses [18], [19]. A high concentration of blood glucose is, therefore, indicative of stress. Furthermore, Nicol et al. [20] found that elevated concentrations of blood glucose were positively correlated with other well-validated indicators of a negative welfare status.

This study aimed to establish whether birds reared in aviaries and producing in furnished cages showed behavioural indicators of poorer welfare than did birds producing in furnished cages after rearing in traditional rearing cages. It was hypothesised that, due to the effect of frustration and stress caused by environmental restriction, birds reared in aviaries would show poorer welfare than those raised in rearing cages. Welfare indicators used were occurrence of comfort behaviours and aversion-related behaviours during undisturbed conditions, alert behaviour in response to a novel object, basal blood glucose concentrations, and mortality. Comfort behaviour is an indicator of good welfare, as its performance declines under conditions conducive to stress and it is associated with positive choice [2], [20], [21]. These behaviours serve the purpose of maintaining the hen's mental and physical wellbeing [22]. Frequency of comfort behaviour is reduced by increased stocking density [23] and spatial restriction [24], increased during anticipation of positive events [25], and modified by social factors [26]. Contrary to comfort behaviours, aversion-related behaviours such as head shaking, self-scratching and short bouts of preening, are associated with negative choice and mild stress and can be interpreted as displacement behaviours [20], [21], [27], [28]. Although traditionally interpreted in relation to fear and anxiety [29], increased performance of alert behaviour directed towards a novel object in the home environment is associated with positive choice and good welfare in laying hens [2], [20]. Comfort behaviour, aversion-related behaviour and alert behaviour towards a novel object, and basal blood glucose concentrations were used to identify differences in the welfare status of the two groups of birds within a commercial production setting. The video observations were taken at 19 and 21 weeks, to ascertain whether any observed differences persisted after the birds should have settled into their new surroundings. We predicted that birds reared in cages would display more comfort behaviour and less aversion-related behaviour under undisturbed conditions, more alert behaviour following introduction to the novel object, and have lower resting concentrations of blood glucose. Any effect of aviary rearing on mortality was predicted to be negative.

## Materials and Methods

### Ethical statement

After reading a detailed formal application for permission to perform this field study (application ID 3868) the Animal Research Authorities (‘Forsøksdyrutvalget’, Norwegian Food Authority, Norwegian Government) stated that no specific permission was needed for the activities and locations involved. The rearer had previously received permission from the Norwegian Food Authority to rear birds in traditional rearing cages. Following the study the birds continued to be housed for egg production purposes until their euthanasia at 76 weeks of age. The study did not involve endangered or protected species. Birds were reared at private facilities with the GPS coordinates 58.704772, 5.650671 and adult birds were housed at private facilities with the GPS coordinates 61.688906, 5.925694.

### Subjects and housing

Non-beak trimmed, female Lohmann-selected leghorn chickens (*Gallus gallus domesticus*) of ages 0–21 weeks and of normal health status were used in this study within a commercial setting. These birds were hatched and reared in one of two rearing treatments: an aviary or in a conventional cage rearing system. All incubating eggs originated from the same flock and were incubated at the same time by the same hatchery. Birds in the two treatments were provided with the same feed but were housed in different rooms containing either aviaries or rearing cages at the same farm. Rearing cages measured 6050 cm^2^ and contained 17 birds per cage (Housing type: Big Dutchman Universa). The flooring in these cages was wire and no bedding was provided. The density of birds in the aviary rearing system (Housing type: Big Dutchman Natura Rearing) was 24 birds/m^2^. The bedding on the floor of the house was sawdust (small dimension wood shavings). Pullets were provided with *ad libitum* access to feed using a chain dispersal system. The feed type was conventional pullet feed produced and sold by Felleskjøpet, Norway. The diets used were ‘oppdrett 1’ for 0–7 week old birds and ‘oppdrett 2’ for 8–17 week old birds. The nutritional content is optimized for layers of this age according to recommendations by Lohmann (Cuxhaven, Germany).

At 16 weeks the birds from both housing systems were transported to a single farm. The housing at the farm was furnished cages (Housing type: AVIPLUS, Big Dutchman- designed for housing 10 hens according to EU requirements), measuring 63×120 cm (7560 cm^2^) and containing between 8 and 9 birds per cage according to Norwegian legislation. A total of 7500 birds, half of which came from each rearing treatment, were included in the study. The composition of a group was not mixed, cages either contained birds reared in conventional rearing cages or birds reared in aviary systems. The furnished cages included access to dust bathing substrate (a small amount of crushed feed in a 1200 cm^2^, oblong litter bath), a nest box, and two perches. The cages were tiered within the house creating three levels of cages, and arranged in four rows. Each row either contained aviary or cage reared birds. The farm operated on a light cycle that was altered according to recommendations by Lohmann. During the period of behavioural observations, the light in the chicken house turned on at 0700 h and turned off at 1600 h. Feed was provided *ad libitum* using a chain dispersal system in a feeding trough at the front of the cage and water was provided *ad libitum* by nipple drinkers (two per cage).

### Methods

The flock at the producing farm was visited on two separate occasions during the laying period, once at 19 weeks and again at 21 weeks. On the first visit, 51 videos were collected over a period of two days between the times of 0900 h and 1400 h. In the second visit, 48 videos were collected within the same time scheme. Cage was used as the statistical unit and 24 cages per treatment per comparison was used as the minimum sample number according to recommendations by Altman [30]. Both visits involved the collection of video footage from a selection of cages. Some of these cages housed birds reared in aviary systems and others housed conventionally reared birds. Hand held cameras (Everio, JVC) mounted on tripods were set up so that the frontal aspect of the cage was filmed. Cages were selected to represent all areas of the house. Different cages were filmed on each farm visit to avoid effects of the first observation upon the second. Two cages from each treatment (4 cages) were filmed concurrently in order to balance the treatments in case of time effects. After recording started the researcher left the house. Ten minutes after filming was started a researcher returned to add the novel objects to the cages. The novel objects used were transparent plastic bottles, hung with a wire attachment on the front bars of the cage so that the bottle was just inside the cage approximately 10 cm from its right boundary. The researcher then left the room containing the birds, and recording continued for a further 10 minutes. Subsequently the researchers returned to remove the novel objects and the cameras and assembled them in a different location within the house. Footage collection continued in this manner until the required amount was obtained.

### Behavioural indicators of comfort and aversion

Observer XT 7.0 software (Noldus Information Technology, Wageningen, The Netherlands) was used for behavioural analysis of the footage. The behavioural analysis was conducted by a single researcher who was blind to the rearing background of the birds. Observations commenced after one minute of recording to avoid recording behaviour of the birds in the presence of the researcher. The observation was continued for 8 minutes subsequently. A focal subject was selected in the following manner: the video was paused at the start of the observation. Chickens were numbered from left to right, and a bird selected randomly. If the focal subject was to move out of view of the camera, the chicken immediately to its right as observed from the camera's viewpoint became the focal subject, and was observed subsequently. If there was more than one chicken to the right of the original focal subject, the bird closest to the front of the cage was chosen. The behaviours noted are presented in Table 1. For preening, bout length was measured as well as frequency and total duration. For the remaining variables, only the frequency was recorded.

**Table 1:** Ethogram of comfort and aversion-related behaviour [20].

Behaviour	Description
Flap wings	Bilateral wing movement including wing raising
Stretch wings	Unilateral backward and downward stretching of leg and wing together
Dust bathe	Lie on side, scratch at cage floor, rub head and neck on floor, open wings
Feather raise	Raise feathers with or without rigorous rotation of body around axial plane, subsidence of feathers back to smooth position
Preen	Raise feathers and clean or realign them with beak
Scratch self	Leg brought upwards and forwards under wing to scratch lowered head
Tail wag	Rapid sideways movement of tail
Shake head	Rapid rotary movement of head, accompanied by slight raising of head and neck feathers

### Alert behaviour

Observations commenced one minute after placement of the novel object into the home cage. The observation was continued for 8 minutes subsequently. Prior to starting observation a focal subject was selected in the same manner as for observations of undisturbed birds. In the event of the focal subject's movement out of view of the camera, the protocol for reselection was to observe the bird in front of the previous focal bird, to avoid influencing the duration of occupation in any given zone (Table 2). Behaviours were coded in such a way that any one code represented the zone of occupation (proximity to novel object), modified to describe the behaviour (or lack thereof) performed at that point. Therefore, all variables were recorded continuously and were mutually exclusive.

**Table 2:** Ethogram of alert behaviours including definitions of proximity to novel object [20].

Behaviour	Description
Near to Novel Object	Subject's head occupies the half of the cage housing the novel object.
Far from Novel Object	Subject's head occupies the half of the cage farthest from the novel object.
Alert Behaviour	Neck extended vertically, either eye oriented towards the novel object. Includes alert behaviour in both sitting and standing positions, but sitting as a component of nesting or dust bathing not included [20]. Extended neck behaviour for the purpose of drinking not included.

### Physiological data

Also at weeks 19 and 21, blood glucose concentrations were measured on the final day of behavioural observation after all behavioural observations were completed. Sampling was performed in the following manner for all birds: one hen from each of 12 cages per row was sampled, six cages on either side of the row, two cages on each tier (bottom, middle or top) resulting in a sample size of 24 per treatment per visit (n = 24). Different cages were sampled on successive visits to avoid sampling the same chicken twice, and to exclude possible effects of previous testing. Each bird was caught by one researcher that gently held the bird in an upright position. A drop of blood was then collected on the strip of an Accu-Check Mobile glucose monitor by another researcher after pricking the birds comb with a Haemolance lancet (puncture depth: 1.8 mm). Values were read directly from the monitor. The duration of the procedure from collection of the bird to removal of blood was ≤ 1 min.

### Production data

Production data were collected by the producer and are summarized for 20, 24, 28, 41 and 73 weeks of age. These data included egg production, average egg weight and egg quality, illustrated by the number of eggs with hairline cracks. Average egg weight data were calculated by the producer after weighing 720 eggs per treatment at each time point. Hen mortality was noted throughout the production period until euthanasia at 76 weeks of age.

### Statistical analysis

All statistical analysis was performed using JMP version 9.0 (SAS Institute Inc., NC, USA). Comfort behaviour was comprised of long bouts of preening (over 2 seconds long), wing flapping, wing stretching, dustbathing, feather raising, and tail wagging. Aversion-related behaviour was comprised of short bouts of preening (up to 2 seconds long), self-scratching and head shaking. Neither comfort behaviour nor aversion-related behaviour conformed to the assumptions of the general linear model (GLM; normal distribution of residuals, equality of variance and linearity). This was related to the fact that a large number of birds showed no comfort or aversion-related behaviour. Therefore, a new ordinal variable was created to indicate whether a bird showed comfort or aversion-related behaviour or not, and this variable was used for analysis. The effect of treatment on the number of birds showing comfort behaviour and/or aversion-related behaviour was then analysed using ordinal logistic regression in a model including effects of rearing treatment, cage height (bottom, middle or top) and the interaction between treatment and cage height. The appropriateness of the model was confirmed using a receiver operating characteristics (ROC) diagram. The ROC diagram, assessing both treatments simultaneously, produced an area under the curve of 0.77 for comfort behaviour for birds at 19 weeks of age and 0.66 at 21 weeks of age. The corresponding numbers were 0.66 for aversion-related behaviour for birds at 19 weeks of age and 0.59 at 21 weeks of age, indicating that all four models were a good representation of the data. Results for ordinal data were analysed using a chi-square test (logistic analysis) and are presented as chi-squared values with corresponding p-values.

Long and short preening behaviour was also analysed separately. The threshold of two seconds, to differentiate between short preening and long preening was chosen according to work done by Duncan and Woodgush [21] indicating that displacement preening bouts are around one to two seconds long. Similar to the other comfort and aversion-related behaviours, preening did not conform to the assumptions of the GLM due to the large number of animals not performing any preening. Therefore, an ordinal variable was also created for this data set, indicating whether the birds performed long and/or short preening or not. The same modelling procedure as explained above was performed. The area under the ROC curve was 0.85 for short preening for birds at 19 weeks of age and 0.69 at 21 weeks of age, and 0.63 for long preening at 19 weeks of age and 0.64 at 21 weeks of age, indicating that all four models were a good representation of the data.

The duration of alert behaviour performed in the half of the cage closest to the novel object conformed to the assumptions of the GLM. We, therefore, tested effects of rearing and cage height, as well as the interaction between these factors on the duration of alert behaviour using ANOVA. Results for ANOVA are presented as F-values and p-values, and data for the treatments are presented as LS Mean ± SE. Data for glucose concentrations and weight of chickens at death were normally distributed and the student's t-test was used in both cases to compare rearing treatments. Hen mortality, egg production and egg quality data are reported as chi-squared and p-values. Because eggs were not weighed individually, numerical values are presented as background information without any corresponding statistical analysis.

## Results

A total of 99 birds were recorded over the course of the study for the collection of comfort behaviour and aversion-related behaviour data. Some behaviours listed in the comfort behaviour ethogram, such as wing flapping and dustbathing, were not observed at all. The others had low frequencies; wing stretch, for example, was only observed four times. In general, comfort behaviour was performed by 80 of the total of 99 birds recorded for both visits. Aversion-related behaviour, the ordinal variable derived from the combination of short preening, head shaking and scratching, was only performed by 41 of the 99 birds. Similarly, short preening was only observed in 9 of the birds, whereas long preening was observed in 41 birds.

The models for comfort behaviour, aversion-related behaviour, short preening and long preening bouts had five degrees of freedom. The number of birds demonstrating comfort behaviour at 19 weeks had a tendency to be influenced by the interaction between treatment and cage height, where aviary reared birds tended to perform more comfort behaviour in the lower cage than cage reared birds (whole model: χ^2^ = 10.05; n = 51; *P* = 0.07; effect of interaction between treatment and cage height: χ^2^_df = 2; n = 51_  = 5.77; *P* = 0.06). However, the ordinal logistic regression model was not significant at 21 weeks (χ^2^ = 3.74; n = 46; *P* = 0.59). The model for aversion-related behaviour was not significant at 19 weeks of age (whole model: χ^2^ = 5.60; n = 53; *P* = 0.35) or at 21 weeks of age (whole model: χ^2^ =  1.50; n = 46; *P* = 0.92).

The number of birds demonstrating short preening at 19 weeks of age had a tendency to be influenced by the cage height, with significantly fewer birds housed in cages on the third cage performing bouts of short preening (whole model: χ^2^  = 9.36; n =  = 53; *P* = 0.10; effect of cage height: χ^2^ = 6.02; n = 53; *P* = 0.05). The model was not significant at 21 weeks of age (χ^2^ = 3.35; n = 46; *P*  = 0.65). The ordinal logistic regression for long preening was insignificant at 19 weeks (χ^2^ = 3.16; n = 53; *P* = 0.68) of age and at 21 weeks of age (χ^2^ = 3.56; n = 46; *P* = 0.61). The model testing for treatment effects on the duration of time spent alert in the half of the cage closest to the novel object at 19 weeks of age was significant (F_(5,44)_ = 2.65; *P* = 0.04). Aviary reared birds spent more time showing alert behaviour (80.93±9.64 sec) than cage reared birds (40.85±10.91; *P* = 0.01). The model at 21 weeks of age was not significant (F_(5,42)_ = 0.36; *P* = 0.87). At 21 weeks of age aviary reared birds were alert for 28.50±6.03 sec and cage reared birds were alert for 29.18±5.91 sec.

There was no significant difference in blood glucose levels between the rearing treatments at 19 (T = -0.159; df = 45.56; *P* = 0.87) or 21 weeks of age (T = 0.065; df = 44.20; *P* = 0.95). Aviary reared birds had significantly higher mortality than cage reared birds (χ^2^_df = 1; N = 303_ = 45.46; *P*<0.001) with 209 dead aviary birds compared with 94 dead cage birds throughout the production period. The birds were transferred to the production farm in late April 2012 and therefore were 21 weeks old at the end of May. Aviary birds had higher mortality than cage reared birds in June (χ^2^ = 14.47; *P*<0.001), July (χ^2^ = 5.63; *P* = 0.02), August (χ^2^ = 6.36; *P* = 0.01), September (χ^2^ = 6.29; *P* = 0.01), October (χ^2^ = 8.03; *P* = 0.01), November (χ^2^ = 4.05; *P* = 0.04), and January (χ^2^ = 4.80; *P* = 0.03). The chickens reached 76 weeks of age in May 2013. However, there was no significant difference in the weight of dead birds between the treatments (T = 0.68; df = 64.98; *P* = 0.49). Aviary reared birds produced a higher number of eggs during the first two months of the laying period compared to cage reared birds (20 weeks: χ^2^ = 711.95; *P* = 0.001; 24 weeks: χ^2^ =  9.24; *P* =  0.002). However, at 28 and 41 weeks, cage reared birds produced more eggs than aviary reared birds (28 weeks: χ^2^ = 5.05; *P* = 0.03; 41 weeks: χ^2^ = 5.23; *P* = 0.02). There was no difference in number of eggs produced at 73 weeks of age (χ^2^ = 1.017; *P* = 0.31). A tendency towards significance was found at 28 weeks of age for the number of eggs with hairline cracks between the treatments (χ^2^ = 3.01; *P* = 0.08), with aviary birds producing 0.34% (12 out of 3570) eggs with cracks compared to 0.14% (5 out of 3640) from cage reared birds. No significant difference was found at any of the other time points (*P*>0.05). Table 3 summarizes the data on average egg weight from each treatment at each time point.

**Table 3:** Average egg weight and total egg production on a given day for aviary and cage reared birds at 20, 24, 28, 41 and 73 weeks of age.

	Average egg weight (g)		Total production (kg)	
Age of hens (weeks)	Aviary reared	Cage reared	Aviary reared	Cage reared
**20**	46.16	46.06	90.01	38.23
**24**	53.24	53.84	172.50	170.03
**28**	58.36	60.65	209.04	221.07
**41**	61.42	63.77	216.26	231.55
**73**	64.68	66.43	200.90	214.90

## Discussion

Contrary to our predictions, aviary reared hens were observed to perform a longer duration of alert behaviour, compared to cage reared hens. In addition, blood glucose concentrations did not differ between rearing treatments, once more in contradiction to our predictions that aviary reared birds would be more stressed than cage reared birds after transfer to furnished cages. Conversely, the negative effects of aviary rearing on mortality did support our expectations. These results paint a complex picture of rearing effects on welfare during production in furnished cages. In general, the performance of comfort behaviour was low and some specific behaviours, namely dust bathing and wing flapping, did not occur at all. Similarly, aversion-related behaviours were also rare. Nevertheless, contrary to our predictions, no significant effects of rearing environment on the performance of comfort behaviours or aversion-related behaviours were found.

The longer duration of alert behaviour by aviary reared hens at the third week after transfer suggests that these hens have better welfare than cage reared hens. Aviary rearing exposes the birds to a greater number of novel situations on a daily basis. The aviary environment presents a larger space to explore and more conspecifics to interact with. Furthermore, the greater space available to the hens allows them to escape situations they would rather avoid. Chronic stressors that cannot be predicted or avoided generally result in depression-like symptoms referred to as learned helplessness, which is normally characterized by a lack of responsiveness to external stimuli [28], [31], [32]. Freedom of movement in aviary reared hens is likely to provide them with an experience of having control over their surroundings, which again would reduce the risk of developing learned helplessness. Learned helplessness was not directly measured in the present study, however it may be related to the lower alert behaviour performance by cage reared birds compared to aviary reared birds. Viewing the results in light of the well-validated interpretation described by Nicol et al. [2], these behavioural observations indicate that aviary reared birds demonstrate a better capability to cope with environmental change than cage reared birds, and experience better welfare, at least during the first period after transfer from rearing to production environment.

Blood glucose concentration is used as an indicator of stress as it increases as a result of corticosterone secretion from the adrenal cortex following activation of the hypothalamic-pituitary-adrenocortical (HPA) axis. Low blood glucose concentrations have also been validated as an indicator of welfare based on their negative association with positive choice [20]. In the current study, no difference between the blood glucose concentrations of aviary and cage reared birds was found. This was contradictory to our prediction that aviary reared birds would be frustrated following transfer to the more spatially restricted and less enriched environment. These results do not completely support the findings from the behavioural observations which indicate that aviary reared birds have better welfare than cage reared birds at the third week following transfer between systems. It is, however, likely that behaviour is a more sensitive measure of the birds' response to environmental change than activation of the HPA-axis.

In this study, mortality was higher in aviary reared hens (5.52%) compared to cage reared hens (2.48%) throughout the laying period. With an average national mortality rate of 2.82% of the flock, the results of this study clearly show that aviary reared birds were more than twice as likely as cage reared birds to die whilst producing in furnished cages. This higher mortality observed in aviary reared hens introduces the question of why these birds were more susceptible to mortality. A large proportion of the birds that were found dead had bloody sores to the head and neck region indicative of injurious pecking. This suggests that the aviary reared birds housed under the present conditions may have been more susceptible to the development of injurious pecking than cage reared birds. Recent studies suggest that experience with litter that later becomes unavailable may increase the frequency of feather pecking in laying hens relative to situations in which birds with no access to litter are later given access [4], [33]. Furthermore, it has been shown that hens exhibit increases in frustration-induced pecking towards conspecifics as a consequence of thwarting access to a reward or expected resource [26]. Feather pecking and injurious pecking are associated with cannibalism [34]. On this basis, one could suggest an explanation of why aviary reared birds appeared to be more susceptible to mortality than their cage reared counterparts following transfer to furnished cages. The light intensity inside the producing farm barn was kept between 10 and 15 lux during the laying period, as recommended by Lohmann. Perhaps these values are above the optimum for birds that are reared in aviaries. It has been suggested that a light intensity of 5 lux is adequate for laying hens [35], [36]. However, there is little evidence that high light intensities are the cause of feather pecking. Indeed, a previous study showed that 10-15 lux does not cause more feather pecking than 3-5 lux in a free-range system [37]. Future research would be necessary to illuminate potential interactions between rearing and housing conditions.

The results from the present study illustrate the complexity of factors influencing welfare, health, and productivity in laying hens. The behavioural data indicate that aviary rearing produces more resilient animals that are better able to cope with and adapt to changes in the environment, probably resulting in better welfare at least at the third week after transfer from the rearing farm to the producing farm. The data on mortality, on the other hand, suggest the contrary. The contradiction between behavioural and mortality data might be explained by changes over time. Performance of alert behaviour by aviary reared birds decreased from week 19 to week 21, and may have continued to do so throughout the rest of the laying period. A longer-term longitudinal study could be used to shed light on this possibility. Furthermore, in addition to monitoring behavioural indicators of welfare throughout the laying period, further investigation of the welfare of cage reared hens could be done with a reversed version of the current study, where both aviary and cage reared hens were transferred to aviaries after rearing.

In addition, further research into the interpretation of specific behaviours is necessary, for example preening behaviours. The threshold of two seconds chosen for the current study was based on research by Duncan and Woodgush [21]. They examined preening behaviour in video recorded chickens, frame by frame. The mean preening bout length in a frustrating situation was 0.9 seconds, compared to 1.33 seconds during a control situation. Their control treatment was also a test condition in a different test room and in isolation from conspecifics, whereas the videos of the present paper were taken in the home cage, arguably a context causing less stress. Nevertheless, their report suggests that the cut-off of two seconds used in the present study should be a valid threshold for discriminating between preening durations reflecting positive and negative valence.

In the present study, aviary reared birds produced more eggs for the first two months of the laying period, but were later surpassed by cage reared birds. There was no overall effect of treatment on the number of cracked eggs, however at week 28 there was a trend towards a higher number of cracked eggs from aviary reared birds. Furthermore, while no statistical testing could be performed, there was a numerical difference in the average weight of eggs between the treatments for ages 28, 41 and 73 weeks, with aviary reared birds laying lighter eggs than cage reared birds. As already mentioned, there are previous reports that cage reared hens produce heavier eggs [7] and floor reared hens produce a higher frequency of dirty and cracked eggs [8]. Taken together these results suggest that environmental conditions influence sexual maturation, rate of lay, and egg size. Another experimental study also suggests that loose housing stimulates the reproductive system as indicated by an increased concentration of sex steroids by laying hens that were loose housed compared to hens housed in cages [38]. In this study, the producers were initially asked to register the occurrence of calcium and blood spots on eggs collected from the aviary and cage reared birds, as these may increase with increasing stress in the hens [39]. Systematic registrations were however stopped early in the study because nearly no calcium or blood spots were observed for either treatment.

The question of rearing effects on resilience, robustness and, ultimately, welfare could also be approached through closer investigation of the brain. Behavioural parameters are ideal for the purpose of welfare measurement, and based on the present results appear at least to be more sensitive than measures related to activation of the HPA-axis, as they are the result of a sum of various internal and external factors, readily altered in response to affect and readily measurable using stringent observation. However, more specific analysis of biochemical and molecular parameters may provide answers as to how the behavioural effects were caused. For example, dopamine turnover has been shown to be positively associated with feather pecking [40], [41]. Furthermore, increase in spatial complexity of housing environment from 16 weeks of age has been shown to result in a left asymmetry in the dopaminergic system in the dorsomedial hippocampus of laying hens [42], suggesting that alterations to this system may underlie some of the effects observed in the current study.

In summary, observations of alertness towards a novel object indicated that aviary reared birds had better welfare than cage reared birds at the third week following transfer from the rearing to the production environment. This may suggest an increased ability to cope with environmental change. However, the higher mortality of aviary reared birds suggests that their later welfare may be compromised. These findings preclude the possibility of drawing general conclusions regarding which rearing method is most suitable for ensuring the welfare of hens destined to produce in furnished cages.
